# Cost-effectiveness of transplanting lungs and kidneys from donors with potential hepatitis C exposure or infection

**DOI:** 10.1038/s41598-020-58215-z

**Published:** 2020-01-29

**Authors:** Nick Scott, Greg Snell, Glen Westall, David Pilcher, Michelle Raggatt, Rowan G. Walker, Margaret Hellard, Anton Y. Peleg, Joseph Doyle

**Affiliations:** 1 Disease Elimination Program, Burnet Institute, Melbourne, Australia; 2 Department of Epidemiology and Preventive Medicine, Monash University, Melbourne, Australia; 3 Lung Transplant Service, The Alfred Hospital, Melbourne, Australia; 4 Department of Intensive Care, The Alfred Hospital, Melbourne, Australia; 5 Renal Medicine, The Alfred Hospital, Melbourne, Australia; 6 Department of Medicine, Monash University, Melbourne, Australia; 7 Department of Infectious Diseases, The Alfred Hospital Central Clinical School, Monash University, Melbourne, Australia; 8 Infection and Immunity Theme, Monash Biomedicine Discovery Institute, Department of Microbiology, Monash University, Clayton, Australia

**Keywords:** Hepatitis C, Translational research

## Abstract

Organ transplant guidelines in many settings recommend that people with potential hepatitis C virus (HCV) exposure or infection are deemed ineligible to donate. The recent availability of highly-effective treatments for HCV means that this may no longer be necessary. We used a mathematical model to estimate the expected difference in healthcare costs, difference in disability-adjusted life years (DALYs) and cost-effectiveness of removing HCV restrictions for lung and kidney donations in Australia. Our model suggests that allowing organ donations from people who inject drugs, people with a history of incarceration and people who are HCV antibody-positive could lead to an estimated 10% increase in organ supply, population-level improvements in health (reduction in DALYs), and on average save AU$2,399 (95%CI AU$1,155-3,352) and AU$2,611 (95%CI AU$1,835-3,869) per person requiring a lung and kidney transplant respectively. These findings are likely to hold for international settings, since this policy change remained cost saving with positive health gains regardless of HCV prevalence, HCV treatment cost and waiting list survival probabilities. This study suggests that guidelines on organ donation should be revisited in light of recent changes to clinical outcomes for people with HCV.

## Introduction

The advent of direct-acting antiviral treatments for hepatitis C virus (HCV) has the potential to significantly reduce HCV associated morbidity and mortality, transforming HCV from a deadly chronic disease to something that is easily curable in greater than 90% of patients^1,2^. HCV is transmitted via exposure to infected blood, and in developed countries HCV transmission occurs primarily among people who inject drugs (PWID)^3–6^, but is also reported through unsafe tattooing practices (in particular in prisons^7–9^) and through sexual contact among HIV-positive men who have sex with men^10–13^. As a consequence, people with a lifetime history of injecting drug use or incarceration, as well as people living with HIV, are considered to have been potentially exposed to HCV.

Current Australian organ transplant guidelines are conservative and recommend that people with active HCV infection (HCV nucleic acid test [NAT] positive) should be considered unsuitable to donate organs^14^. In addition, risk stratification is recommended for potential donors who are at-risk of HCV, with many potential donors being rejected due to past infection (i.e. anyone who is HCV antibody positive) or perceived concerns due to the potential donor being at risk of acquiring HCV. This is a risk-adverse approach, as past or potential exposure to HCV does not necessarily reflect HCV status, and comes from an era when highly-effective HCV treatments were not available and clinical outcomes for people living with HCV were far less optimistic^15^. Clinical practice has now changed considerably and there is a need to revisit exclusion criteria for HCV based on contemporary information. Increasingly donors with past HCV infection (HCV antibody positive but NAT negative) are being considered for willing and informed recipients, and some centres are piloting lung and kidney donations from those with active infection (HCV NAT positive)^16^. The likelihood of HCV transmission through transplantation and the associated risks and health costs must be balanced against the lost opportunity of not using organs for transplantation to terminally ill patients.

Organ transplant waiting lists are notoriously long and many people die while waiting to receive a life-saving donation^17^. This is primarily due to the small proportion of people who die in the specific circumstances under which organ donation is medically feasible (approximately 1% of hospital deaths^14^). Therefore when these circumstances are met, maximising the supply of organs by minimising exclusion criteria is critical. For example, in Australia 718 kidneys and 375 lungs were transplanted in 2015^18^; however eight people died waiting for kidney transplants, nine people died waiting for lung transplants^17^, 12,706 people were on dialysis^19^ and 90 people remained on the waiting list for a lung transplant^20^. In the same year approximately 10% of potential donors were excluded based on potential exposure to HCV^21^ (approximately 38 and 72 lung and kidney transplants respectively, based on 2015 donor numbers^18^), indicating that relaxation of this exclusion criterion could potentially have an immediate impact on the quality of life for those on the waiting list.

The risks of transplanting an organ from a HCV infected source is in causing new HCV in the organ recipient. If HCV were transmitted to a recipient through a transplant, and the recipient failed to clear HCV through treatment, HCV in combination with lifelong transplant-related immunosuppressing medications would accelerate the progression of HCV-related liver disease^22,23^. Under these circumstances the individual patient may have had better long-term health outcomes had they spent a longer period on the waiting list to avoid an HCV-infected organ.

In order to consider whether removing the current policy, which deems anyone with potential HCV exposure, past HCV infection or current HCV infection (PWID, people with a history of incarceration and anyone who is HCV antibody positive, regardless of HCV NAT status—henceforth people with “potential HCV infection”) unsuitable to donate, there is a need to weigh the potential gains from increased organ supply (leading to reduced deaths on the waiting list and less time spent in a state of poor quality of life) against the potential negative losses (accelerated liver disease and mortality risks for those who become HCV-infected following an organ donation and fail to respond to HCV antiviral treatment). In light of the HCV antivirals’ potential to clear HCV from infected people, this paper uses a mathematical model to determine the cost-effectiveness of removing potential HCV infection as an exclusion criterion for organ donation. We estimate the expected change in average healthcare costs and disability-adjusted life years (DALYs) per person associated with the policy change in Australia, and perform a sensitivity analysis around model inputs to make the results applicable to other international settings.

## Methods

**Figure 1:**
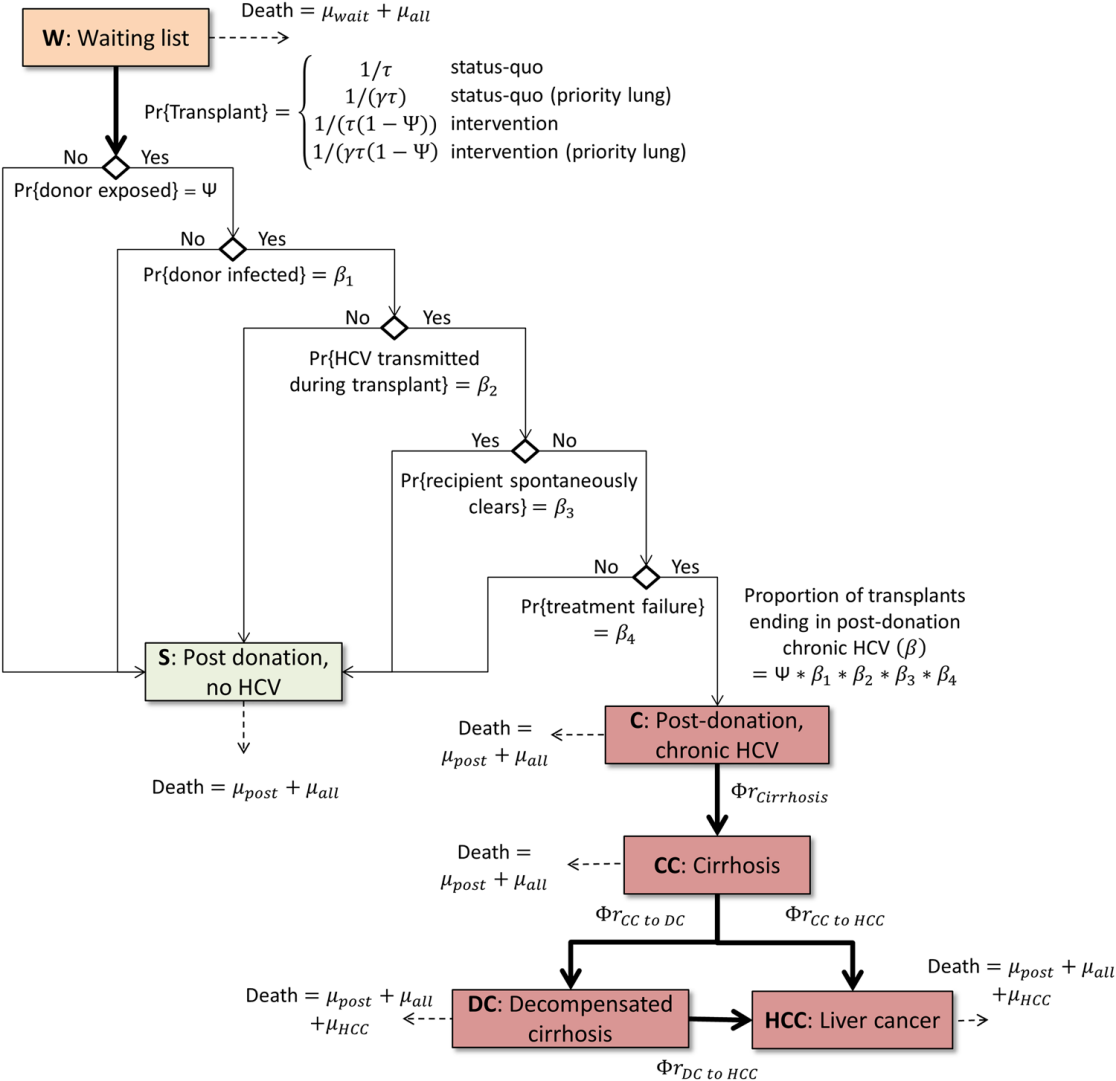
Model schematic. The model was initiated with a cohort of individuals waiting to receive an organ. People in the model could die while on the waiting list, from complications after receiving a transplant or from age-dependent all-cause mortality. When past or current HCV infection was removed as a donor exclusion criterion, transplant waiting times were decreased and individuals could end up chronically infected post-transplant if: the donor had been exposed, the donor was chronically infected, the infection was transmitted during transplant and the recipient failed to spontaneously clear or respond to prophylactic HCV treatment. People who ended up with a chronic HCV infection post-transplant could develop advanced liver disease, increasing their mortality risks.

A deterministic cohort model was used (Fig. 1), with independent copies and alternate parameters used for people requiring lung and kidney transplants. All parameters, their uncertainty ranges and sources are provided in Table 1. The model was implemented in Matlab 2018b and progressed in weekly time steps (where applicable annual transition probabilities in Table 1 were converted to weekly transition probabilities by inverting the formula p_annual_ = 1 − (1-p_weekly_)^52^).

**Table 1:** Model parameters. Parameters and uncertainty ranges related to the waiting lists, organ donation, HCV transmission, disease progression and mortality.

Waiting list parameters	Symbol from text and equations	Best estimate	Range	Ref.
Age of people on waiting list	λ	Lung: 55 years Kidney: 52 years	Lung 5–73 Kidney 1–76	Kidney: transplant waiting list^31^. Lung: private communication.
Proportion of potential lung recipients deemed high priority	W_high_	25%	±10%^a^	Expert opinion.
Annual probability of death while on waiting list	μ_wait_	Lung (regular): 2.8% Kidney: 14%	±10%	Lung (regular): private communication of waiting list outcomes. Kidney: in 2016 there were 12,706 patients on dialysis and 1,764 deaths^17,19^. Note there is some uncertainty as only approximately 1/3 of dialysis patients are on the wait list.
Increased mortality risk for people on the priority lung waiting list	Γ	10.7	±10%	Based on an annual probability of death while on waiting list of 30% (expert opinion). Therefore 10.7 = 30/2.8 from above. Tested in sensitivity analysis.
Average duration on wait list	τ	Lung (regular): 180 days Lung (priority): 60 days Kidney: 2.4 years	Lung: 0–3.5 years Kidney: 1.4–4.4 years	Lung (regular): expert opinion. Lung (priority) and kidney: median and inter-quartile range from waiting lists^20,24^.
**Donor parameters**				
Percentage of useable lung donations that can be considered for a high priority patient	1-γ	50%	±10%	Expert opinion. Donor lungs are targeted to the priority group with roughly half being compatible.
Percentage of potential donors classified as having potential HCV infection (therefore currently screened out)	Ψ_1_	Lung: 10% Kidney: 10%	±10%	^21^
**Post-transplant parameters**				
Annual probability of death post-transplant	μ_post_	Lung: 8.9% Kidney: 2.1%	±10%	Lung: five-year survival of 62.6%^20,25^. Calculated by 0.626 = (1-p)^5^ Kidney: five-year survival 90%. Calculated by 0.90 = (1-p)^5^
**Intervention**				
Percentage of donors with potential HCV infection who would be screened out due to other factors anyway (e.g. smoking)	Ψ_2_	Lung: 31.9% Kidney: 5.6%	±10%	Lung: 240/752^32,33^. Kidney: 46/824^32,33^. Both assumed equivalent to percentage of organs deemed ‘not medically suitable’.
**HCV transmission**				
Percentage of donors with potential HCV infection who actually have HCV (HCV NAT-positive)	β_1_	6.6%	±10%	HCV prevalence in ‘high risk behaviour’ organ donors^34^. Assumed same for lungs and kidneys.
Probability of acquiring HCV from a HCV-infected donor organ	β_2_	94%	86–100%	^35–37^. Assumed same for lungs and kidneys.
Probability of spontaneously clearing HCV if infected from a transplant	1-β_3_	0%	0–29%	^38^. Conservative estimate in the setting of immunosupression.
HCV cure rate post-transplant	1-β_4_	95%	±10%	^39,40^.
**HCV parameters**				
Relative increase in liver disease progression post-transplant	Φ	27 times	6–40 times	OR = 70.1 (95%CI 6.4–770) for fibrosis progression in patents with HCV infection post-transplant compared to HCV before transplant^22^. Let ${p}_{pre},\,{p}_{post}$ be the pre and post annual probability of developing cirrhosis. Then $OR=\frac{{p}_{post}}{1-{p}_{post}}/\frac{{p}_{pre}}{1-{p}_{pre}}$ and ${p}_{post}=OR{p}_{pre}/(OR{p}_{pre}+1-{p}_{pre})\,$If ${p}_{pre}=2.4{\rm{ \% }}$ (see below), then ${p}_{post}=64.0 \%$, giving a factor of 27 increase in the *rate* of progression.
Annual probability of developing cirrhosis	r_Cirrhosis_	2.4%	SD = 0.025	Combination weight from the annual disease stage progressions: 10.6% F0- > F1; 7.4% F1- > F2; 10.6% F2- > F3; 11.1% F3- > F4^41,42^.
Annual probability of developing DC from cirrhosis	r_CC to DC_	3.7%	SD = 0.016	^42^
Annual probability of developing HCC from cirrhosis	r_CC to HCC_	1.0%	SD = 0.007	^42^
Annual probability of developing HCC from DC	r_DC to HCC_	6.8%	SD = 0.015	^42^
Annual probability of death from DC stage	μ_DC_	13.8%	SD = 0.032	^42^
Annual probability of death from HCC stage	μ_HCC_	60.5%	SD = 0.033	^42^
**Annual probability of death from all-cause mortality (per 1000 person years)**				
5–20 year olds	μ_all_	0.010		Assumed to equal the general Australian population. Values from Victoria life tables^26^.
20–24 years old		0.022		
25–29 years old		0.023		
30–34 years old		0.038		
35–44 years old		0.069		
45–54 years old		0.168		
55–64 years old		0.381		
65–74 years old		0.970		
75–84 years old		3.231		
85+ years old		13.337		

Unless explicitly mentioned, parameters relating to the regular and priority lung transplant lists are taken to be equal. ^a^Where uncertainty ranges were not available ±10% was assumed.

### Status-quo

In the status-quo scenario, people in the model were classified as either on the transplant waiting list (W) or post-donation with no HCV (S) (Fig. 1, with the probability that the donor was potentially exposed set to zero). The model was initiated with a cohort of people aged λ on the waiting list, and each time step a proportion 1/τ were assumed to receive an organ and move to the S compartment (with τ estimated as the median waiting period^20,24^).

Mortality was also included. Each time step a proportion of people on the waiting list (μ_wait_)^17,19^ and who had received a transplant (μ_post_)^20,25^ were assumed to die from complications, in addition to a proportion of the whole cohort (μ_all_) who were assumed to die from age-dependent all-cause mortality^26^ (modelled to increase over time as the average age of the cohort increased).

Unlike people on the kidney waiting list who have a more homogeneous mortality risk, there are a subset of people on the lung waiting list who are identified clinically as having a significantly higher mortality risk and are prioritised to receive donor lungs. Therefore, the waiting list in the lung model was stratified by risk, with a subset of patients classified as high priority (W_high_). These high priority patients were modelled to have a higher waiting list mortality (by a factor Γ relative to the low priority group) and shorter waiting time (by a factor γ) due to compatible lungs being preferentially allocated to them.

### Policy change scenario

The intervention being considered enabled people with potential HCV infection to donate organs. This was modelled by reducing the organ waiting time and introducing a risk to patients of acquiring chronic HCV post-transplant.

The waiting period τ was scaled by a factor (1-Ψ), where Ψ represents the proportion of potential organs in Australia that are currently ruled out *uniquely* by these criteria. Ψ was calculated as the proportion of donors that are currently deemed ineligible due to potential HCV infection (Ψ_1_) multiplied by the proportion who would not be ruled out for other factors, such as smoking (1-Ψ_2_).

The proportion of transplant recipients who acquired chronic HCV post-transplant (β) was estimated based on: the probability that the donor was among this newly eligible group (Ψ); the probability that the donor had current HCV (HCV NAT-positive) (β_1_); the probability that the infection was transmitted during transplant (β_2_); the probability that a newly acquired HCV infection would not spontaneously clear in the organ recipient (β_3_); and the probability that a chronically infected organ recipient would fail to be cured from HCV treatment (given early to recipients of HCV NAT-positive organs) (β_4_). That is, β = Ψ* β_1_ *β_2_ *β_3_ *β_4_.

In the intervention scenario, the model was again initiated with a cohort of people aged λ on the waiting list. Each time step a proportion (1- β)/(τ(1- Ψ)) were assumed to receive an organ and move to the post-donation no HCV (S) compartment, and a proportion β/(τ(1- Ψ)) were assumed to receive an organ and move to the post-donation chronic HCV compartment (C). Each time step a proportion of people with chronic HCV (Φr_Cirrhosis_) could develop compensated cirrhosis (CC), where r_Cirrhosis_ was the disease progression probability obtained from the literature and Φ > 1 was a factor to account for additional disease progression risks among the post-transplant population. Similarly, a proportion of people with CC could develop decompensated cirrhosis (DC) (Φr_CC to DC_) or hepatocellular carcinoma (HCC) (Φr_CC to HCC_), and a proportion of people with DC could develop HCC (Φr_DC to HCC_). Increased mortality risks were included for people with DC and HCC (μ_DC_, μ_HCC_).

### Model equations

The model scenarios can be described by the following sets of differential equations, based on the parameters and compartments introduced in the above sections. For the kidney status-quo scenario:

$$\frac{dW}{dt}=-\,\frac{1}{\tau }W-({\mu }_{wait}+{\mu }_{all}(t))W$$

$$\frac{dS}{dt}=\frac{1}{\tau }W-({\mu }_{post}+{\mu }_{all}(t))S$$

For the kidney intervention scenario:

$$\frac{dW}{dt}=-\,\frac{1}{\tau (1-\Psi )}W-({\mu }_{wait}+{\mu }_{all}(t))W$$

$$\frac{dS}{dt}=\frac{1-{\rm{\beta }}}{\tau (1-\Psi )}W-({\mu }_{post}+{\mu }_{all}(t))S$$

$$\frac{dC}{dt}=\frac{{\rm{\beta }}}{\tau (1-\Psi )}W-\Phi {r}_{cirrhosis}C-({\mu }_{post}+{\mu }_{all}(t))C$$

$$\frac{dCC}{dt}=\Phi {r}_{cirrhosis}C-(\Phi {r}_{CCtoDC}+\Phi {r}_{CCtoHCC})CC-({\mu }_{post}+{\mu }_{all}(t))CC$$

$$\frac{dDC}{dt}=\Phi {r}_{CCtoDC}CC-\Phi {r}_{DCtoHCC}DC-({\mu }_{post}+{\mu }_{DC}+{\mu }_{all}(t))DC$$

$$\frac{dHCC}{dt}=\Phi {r}_{CCtoHCC}CC+\Phi {r}_{DCtoHCC}DC-({\mu }_{post}+{\mu }_{HCC}+{\mu }_{all}(t))HCC$$

For the lung status-quo scenario:

$$\frac{d{W}_{high}}{dt}=-\,\frac{1}{\gamma \tau }{W}_{high}-(\Gamma {\mu }_{wait}+{\mu }_{all}(t)){W}_{high}$$

$$\frac{d{W}_{low}}{dt}=-\,\frac{1}{\tau }{W}_{low}-({\mu }_{wait}+{\mu }_{all}(t)){W}_{low}$$

$$\frac{dS}{dt}=\frac{1}{\gamma \tau }{W}_{high}+\frac{1}{\tau }{W}_{low}-({\mu }_{post}+{\mu }_{all}(t))S$$

For the lung intervention scenario:

$$\frac{d{W}_{high}}{dt}=-\,\frac{1}{\gamma \tau (1-\Psi )}{W}_{high}-(\Gamma {\mu }_{wait}+{\mu }_{all}(t)){W}_{high}$$

$$\frac{d{W}_{low}}{dt}=-\,\frac{1}{\tau (1-\Psi )}{W}_{low}-({\mu }_{wait}+{\mu }_{all}(t)){W}_{low}$$

$$\frac{dS}{dt}=\frac{1-{\rm{\beta }}}{1-\Psi }(\frac{1}{\gamma \tau }{W}_{high}+\frac{1}{\tau }{W}_{low})-({\mu }_{post}+{\mu }_{all}(t))S$$

$$\frac{dC}{dt}=\frac{{\rm{\beta }}}{1-\Psi }(\frac{1}{\gamma \tau }{W}_{high}+\frac{1}{\tau }{W}_{low})-\Phi {r}_{cirrhosis}C-({\mu }_{post}+{\mu }_{all}(t))C$$

$$\frac{dCC}{dt}=\Phi {r}_{cirrhosis}C-(\Phi {r}_{CCtoDC}+\Phi {r}_{CCtoHCC})CC-({\mu }_{post}+{\mu }_{all}(t))CC$$

$$\frac{dDC}{dt}=\Phi {r}_{CCtoDC}CC-\Phi {r}_{DCtoHCC}DC-({\mu }_{post}+{\mu }_{DC}+{\mu }_{all}(t))DC$$

$$\frac{dHCC}{dt}=\Phi {r}_{CCtoHCC}CC+\Phi {r}_{DCtoHCC}DC-({\mu }_{post}+{\mu }_{HCC}+{\mu }_{all}(t))HCC$$

### Outcomes

The model was run until everyone in the initial cohort had died. Total costs were calculated by multiplying the person-years spent in each compartment by the annual costs associated with each condition, including medications, procedures and healthcare provider costs (Table 2). DALYs were calculated by adding the years lived with disability (by multiplying the person-years spent in each compartment by the associated disutility weightings in Table 2) to the years of life lost due to deaths (by considering the age at death). Costs, years lived with disability and years of life lost were discounted at 3% per annum^27^.

**Table 2:** Health utilities and costs. Parameters and uncertainty ranges for health utilities and costs. Unless explicitly mentioned, parameters relating to the regular and priority lung transplant lists are taken to be equal.

Health utilities	Best estimate	Range	Ref.
On waiting list	Lung (regular): 0.5 Lung (priority): 0.25 Kidney: 0.5	0.35–0.59	Similar estimates for kidney^43^ and lung^44^. Health utility on priority lung list assumed to be halved.
Post-transplant	0.7	0.56–0.74	Similar estimates for kidney^43^ and lung^44^.
Chronic HCV + post-transplant	0.65	SD = 0.15	^45–47^, assumed same as just HCV.
Cirrhosis + post-transplant	0.55	SD = 0.24	^45–47^, assumed same as just HCV.
Decompensated cirrhosis + post-transplant	0.45	SD = 0.14	^45–47^, assumed same as just HCV.
Hepatocellular carcinoma + post-transplant	0.45	SD = 0.14	^45–47^, assumed same as just HCV.
**Costs**^**a**^			
Annual costs on waiting list	Lung (regular): AU$64,105 Lung (priority): AU$128,210 Kidney: AU$64,105	Lung (regular) and kidney: AU$49,137–79,072 Lung (priority): AU$98,274–158,144	Kidney: Annual cost of dialysis estimated $49,137 (at home) $79,072 (in centre)^48^, median value used. Priority list for lung assumed to incur double the costs due to additional time in hospital and intensive care. Costs are from health system perspective (i.e. does not include loss of income for individuals).
One-off transplant costs	Lung: AU$166,520 Kidney: AU$88,161	Lungs: ±10% Kidney: AU$81,755–94,566	Lung: Includes: Surgery & hospitalisation $122,333; drug costs AU$44,187^48,49^. These costs have reduced significantly since 2014 (making our estimates of cost-effectiveness conservative); however data is not yet available. Kidney: Median cost from surgery & hospitalisation: $37,568 (17 years and over, no serious complications) and $50,379 (less than 17 years, or with CC). Plus drug costs AU$44,187^48,49^.
Post-transplant annual costs	AU$11,770	±10%	Year two onwards costs^48^.
Cost of HCV treatment	AU$1,000	±10%	Tested in sensitivity analysis to vary from AU$0 to AU$20,000. Assumed to be given to all patients receiving a HCV NAT-positive organ.
Chronic HCV + post-transplant	AU$59,389	±10%	AU$44,187 + $15,202, where additional HCV costs are assumed to equal the costs of managing HCV-related cirrhosis in non-transplant patients^50–52^.

^a^AU$1AUD ~ US$0.8.

### Uncertainty analysis

A Monte Carlo uncertainty analysis was conducted to account for multivariate parameter uncertainty. Individual parameters were parametrised as uniform probability distributions, with upper and lower bounds defined by their range as estimated in the literature, or +/−10% of their point estimates where this was not available (Tables 1 and 2). Random sampling was used to generate 1000 parameter sets that were used for 1000 independent model runs. Confidence bounds for costs and DALYs were taken as the interquartile range (IQR) of the resulting 1000 outputs.

### Additional settings

The cost-effectiveness is likely to vary between countries based on current organ waiting times, HCV prevalence among potentially exposed donors, the probability of death while on the waiting list and the cost of HCV treatment. Therefore, to estimate how the results would translate to other settings, a sensitivity analysis was undertaken to test the impact on cost-effectiveness when: the HCV prevalence among potentially exposed donors ranged from 0% to 30%; the cost of HCV treatment ranged from A$0–20,000; the average waiting time on the donor list ranged from 100–200 days on the priority lung waiting list, and 0.5 to 5 years on the kidney waiting list; and the annual probability of death while waiting to receive an organ ranged from 10–20% for kidneys and 10–50% for lungs.

## Results

If a 10% boost in donor numbers were possible by transplanting lungs and kidneys from people with potential HCV infection^21^, this would represent an additional 38 and 72 potentially life-saving lung and kidney transplants in Australia per year respectively (based on 2015 donor numbers^18^).

**Figure 2:**
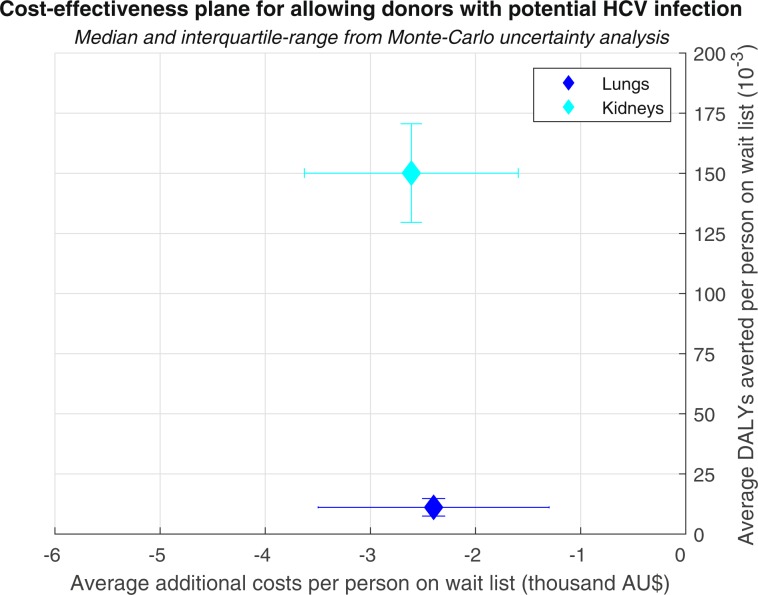
Cost-effectiveness plane for removing potential HCV infection as an ineligibility criterion for lung and kidney organ donations.

Allowing lung and kidney transplants from donors with potential HCV infection was estimated to be cost saving and to result in modestly improved health outcomes (Fig. 2). On average, per person requiring a lung or kidney transplant the policy change saved AU$2,399 (95%CI AU$1,155–3,352) and AU$2,611 (95%CI AU$1,835–3,869) respectively, and averted 0.0111 (95%CI 0.0077–0.0150) and 0.1501 (95%CI 0.1187–0.1597) DALYs respectively. In all cases of the uncertainty analysis, the expected benefits were positive.

### Sensitivity analysis

**Figure 3:**
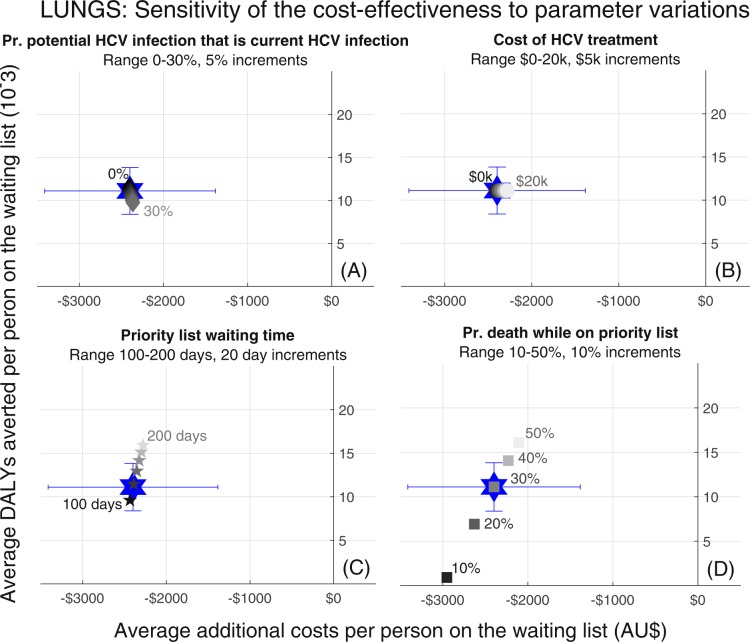
Sensitivity analysis for removing potential HCV infection as an ineligibility criterion for lung transplants. Estimates of the additional costs and DALYs averted per person when parameters are varied from baseline values. (**A**) proportion of donors with potential HCV infection who have current HCV infection; (**B**) cost of HCV treatment; (**C**) average time on waiting list at baseline; (**D**) annual probability of death while on waiting list.

**Figure 4:**
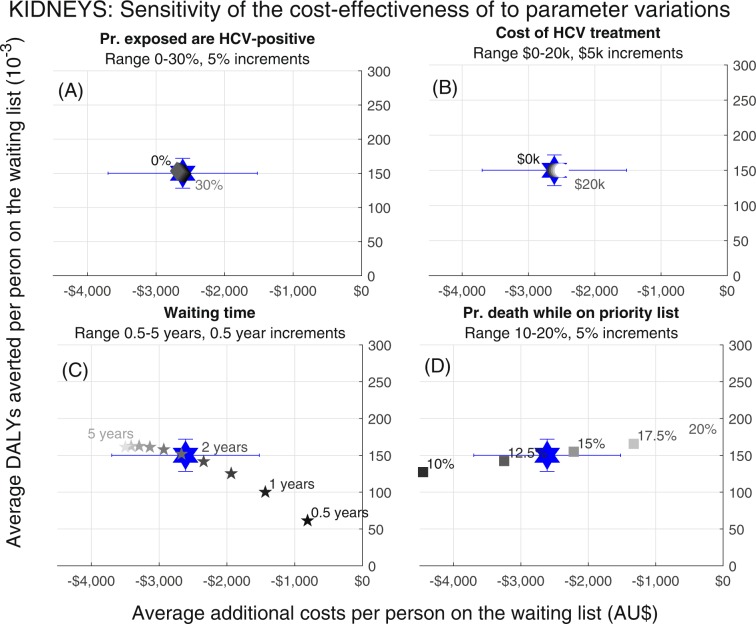
Sensitivity analysis for removing potential HCV infection as an ineligibility criterion for kidney transplants. Estimates of the additional costs and DALYs averted per person when parameters are varied from baseline values. (**A**) proportion of donors with potential HCV infection who have current HCV infection; (**B**) cost of HCV treatment; (**C**) average time on waiting list at baseline; (**D**) annual probability of death while on waiting list.

The policy change to allow donations from individuals with potential HCV infection remained cost saving with positive health gains regardless of HCV prevalence, HCV treatment cost and survival probabilities for people on the waiting lists (Figs. 3 and 4).

The lung transplant model was the most sensitive to changes in the probability of death while on the priority waiting list. When the mortality parameter was lower, fewer DALYs were averted but greater costs were saved, since without the additional organs people on the waiting list were living longer and accruing more costs. The kidney transplant model was similarly sensitive to changes in the probability of death while on the waiting list, as well as to the average time spent on the waiting list (where waiting lists are longer the policy change is more cost saving and averts more DALYs). For both models the cost of HCV treatment had minimal impact since it was always small compared to the costs of care and only given to recipients of HCV NAT-positive donors, (estimated at 6.6% of all those extra organs that might be available under this policy change; Table 1). The background HCV prevalence (i.e. probability of a potential HCV infection being a current HCV infection had minimal impact on results. In particular, the case with zero prevalence represents a scenario where only the benefits of the additional organ supply are seen.

## Discussion

Using a mathematical model we estimated that the inclusion of donors with potential HCV infection for lung and kidney transplantation would be cost saving and result in better overall health outcomes. Whilst calibrated based on Australian inputs, these findings are likely to hold for international settings with comparable health systems, since this policy change remained cost saving with positive DALYs averted regardless of HCV prevalence, HCV treatment cost, donor organ availability and survival probabilities for people on the waiting lists.

Notably, the estimated population-level health impact was quite modest, since it applied to only the relatively small percentage of additional donors–that is, these averages are weighed down by the fact that most people (around 90%) would be unaffected by the change. Individuals who did receive these additional organs would likely have a marked improvement in health and lifestyle, and there is precedence for transplanting organs that need to be followed by therapy, with prophylaxis protocols already in place for many infections (e.g. cytomegalovirus, Epstein-Barr virus, meningococcal meningitis and syphilis).

Because of the extreme benefit possible for these vulnerable patients, there are ethical issues that need to be considered as part of this policy change; individuals on the waiting list would need to be educated about the specific additional risks that apply to them in order to give fully informed consent. There is much excitement about the highly effective treatments for HCV, and as more case studies become available on the outcomes of HCV-infected donor organs, more accurate information about risks of HCV transmission and mitigation will be available for these patients. Nevertheless, initial findings indicate that even in the case that HCV were transmitted, better individual-level outcomes are likely given HCV remains easy to treat post-transplant^16^, in particular if the donor organs are targeted to those most in need.

It is not particularly surprising that the outcomes were robust with respect to the cost of HCV treatment and background HCV prevalence. This is because even for places where HCV infection prevalence (as opposed to merely potential HCV infection) is extremely high, it is only likely to impact a minority of donors; for example Egypt has among the worlds’ highest general population HCV prevalence (approximately 10%^28,29^), but even this represents a minority of donors. Moreover, even for patients who receive an infected organ and fail to spontaneously clear, in places like the US where HCV treatment costs are among the highest globally^30^, this one-off HCV antiviral cost is comparable to the ongoing annual dialysis costs of AU$65,000 for people on the waiting list.

Transplanting HCV-positive lungs and kidneys may be even more cost saving than we have estimated due to a number of conservative assumptions used. First, we have taken a health system approach to estimating costs that does not account for the loss of economic productivity individuals may experience while on the waiting list, or the financial support they may require if unable to work. Second, we considered independent lung and kidney models; however adding a single potentially exposed HCV-infected patient to the donor pool can provide additional organs or tissue for multiple purposes. Third, for more complex cases requiring multiple organs, once a donor-recipient match is found there are likely to be technical efficiency gains through combined procedures. Fourth, and perhaps most importantly, donor organs are allocated according to need and targeted to those most likely to die without them. Therefore, in practice, any additional organs would be provided in a way that maximises the life and health utility gained. For our lung model, we approximated this by including a higher risk category (since the difference between the higher and lower risk is so stark); however the general random mixing assumptions inherent in compartmental models, even within the high-risk category, mean that the benefits of such targeted interventions are underestimated. For our kidney model we have assumed an average disutility while on the waiting list, when in reality these individuals will gradually deteriorate over time, with additional organs being allocated where the benefits are maximised. These underestimates of cost-effectiveness may be balanced in part by the additional HCV testing costs for donors with potential HCV infection, but these are expected to be trivial compared with the costs associated with the ongoing management of patients on the waiting list.

There are several key limitations to this study. Our parameters come from a number of sources with their own uncertainties that may not reflect perfectly the donor and recipient population. Moreover, we have used population-level averages when there is extreme heterogeneity among people requiring lung and kidney transplants. To attempt to account for this uncertainty and heterogeneity, we performed a Monte Carlo uncertainty analysis to incorporate the possible ranges of parameters into our estimates. This resulted in fairly modest confidence intervals, providing confidence in our estimates. Nevertheless, we emphasize that the potential pairing of HCV-infected organs should be carefully considered by specialists before transplant to minimise adverse outcomes. As HCV treatment pilots among organ recipients are scaled-up in the community, it will become clearer which, if any, characteristics may indicate potential HCV treatment failure, and this should form a part of any decision. Our model has also not accounted for the numerous comorbidities that may be present among organ recipients, and again we urge that caution should continue to be taken in the recipient donor matching process.

## Conclusion

In light of recent changes to clinical outcomes for people living with HCV, removing potential HCV infection as an ineligibility criterion for lung and kidney donation would increase the donor pool, be cost saving, and lead to better population-level health outcomes. Guidelines and practice need to be revisited in light of the dramatic change in HCV treatment outcomes, and to determine how to ethically change current practice.

## Data Availability

Data inputs used for the mathematical model are available in Table 1.
